# Evolution of *Burkholderia pseudomallei* in Recurrent Melioidosis

**DOI:** 10.1371/journal.pone.0036507

**Published:** 2012-05-15

**Authors:** Hillary S. Hayden, Regina Lim, Mitchell J. Brittnacher, Elizabeth H. Sims, Elizabeth R. Ramage, Christine Fong, Zaining Wu, Eva Crist, Jean Chang, Yang Zhou, Matthew Radey, Laurence Rohmer, Eric Haugen, Will Gillett, Vanaporn Wuthiekanun, Sharon J. Peacock, Rajinder Kaul, Samuel I. Miller, Colin Manoil, Michael A. Jacobs

**Affiliations:** 1 Department of Microbiology, University of Washington, Seattle, Washington, United States of America; 2 University of Washington Genome Center, University of Washington, Seattle, Washington, United States of America; 3 Department of Genome Sciences, University of Washington, Seattle, Washington, United States of America; 4 Department of Medicine, University of Washington, Seattle, Washington, United States of America; 5 Mahidol-Oxford Tropical Medicine Research Unit, Mahidol University, Bangkok, Thailand; 6 Department of Microbiology and Immunology Faculty of Tropical Medicine, Mahidol University, Bangkok, Thailand; 7 Department of Medicine, University of Cambridge, Cambridge, United Kingdom; Tulane University School of Medicine, United States of America

## Abstract

*Burkholderia pseudomallei*, the etiologic agent of human melioidosis, is capable of causing severe acute infection with overwhelming septicemia leading to death. A high rate of recurrent disease occurs in adult patients, most often due to recrudescence of the initial infecting strain. Pathogen persistence and evolution during such relapsing infections are not well understood. Bacterial cells present in the primary inoculum and in late infections may differ greatly, as has been observed in chronic disease, or they may be genetically similar. To test these alternative models, we conducted whole-genome comparisons of clonal primary and relapse *B. pseudomallei* isolates recovered six months to six years apart from four adult Thai patients. We found differences within each of the four pairs, and some, including a 330 Kb deletion, affected substantial portions of the genome. Many of the changes were associated with increased antibiotic resistance. We also found evidence of positive selection for deleterious mutations in a TetR family transcriptional regulator from a set of 107 additional *B. pseudomallei* strains. As part of the study, we sequenced to base-pair accuracy the genome of *B. pseudomallei* strain 1026b, the model used for genetic studies of *B. pseudomallei* pathogenesis and antibiotic resistance. Our findings provide new insights into pathogen evolution during long-term infections and have important implications for the development of intervention strategies to combat recurrent melioidosis.

## Introduction

Pathogenic bacteria that are able to persist *in vivo* face strong selective pressure during infection leading to variations within clonal lineages. Knowledge of genomic changes in bacteria during prolonged infection is only slowly accumulating [1]. Several studies have focused on comparative genomic analysis of sequential isolates obtained from hosts with chronic infections of *Pseudomonas aeruginosa* [2]–[4], *Helicobacter pylori* [5], [6] and *Staphylococcus aureus* [7]. These studies showed that bacterial adaptation during infections involved, in part, changes in genome sequence or content resulting in enhanced antimicrobial resistance, reduced acute virulence and improved metabolic fitness. Although such changes presumably favor the clonal expansion of the bacteria, the mutations may also create new vulnerabilities that can be exploited for treatment. In contrast to such chronic infections, other pathogenic bacteria, e.g. *Mycobacterium tuberculosis*, can establish long-term colonization with periods during which the bacteria are in a low- or non-replicative state. Non-replicating pathogens are more resistant to current therapeutic approaches and require protracted treatment periods [8]. Despite the need for better intervention strategies, comparative whole-genome analyses of sequential isolates of such persistent bacteria have not been conducted.

*Burkholderia pseudomallei*, the causative agent of the often-fatal disease melioidosis, is capable of causing persistent infections in its human host. This facultative intracellular pathogen can cause acute infections, which if not completely cleared can recrudesce months or years after the initial infection. Recurrent disease in patients who survive a primary episode occurs in approximately 6% in the first year and in 13% within ten years [9]. A study that evaluated recurrent disease in 116 Thai patients in terms of relapse (same strain) or reinfection (different strain) found that 75% of episodes were due to relapse [10]. Latent, asymptomatic infections also occur and may progress to acute melioidosis decades after the initial exposure to the organism [11]–[13]. It is not known if *B. pseudomallei* exists in a dormant state between periods of active disease. The mechanism involved in adaptation to the host niche also is largely unknown [14]. A few studies have focused on bacterial population diversity during the acute phase of *B. pseudomallei* infection [15]–[17], and one report of genetic variation in the *B. pseudomallei* core genome noted a small number of mutational differences between two clonal pairs of isolates [18]; however, to date there have been no investigations focused on comparative genomic analysis of isolates associated with primary and relapse episodes.

The aim of our research was to conduct whole-genome comparisons of clonal primary-relapse *B. pseudomallei* pairs from four adult Thai patients recovered six months to six years apart. For comparison purposes, we included an additional pair isolated during the same primary infection but from different body sites. We completed a base-pair accurate genome sequence for one isolate in this pair, 1026b (GenBank accessions CP002833-CP002834), which has been employed extensively in laboratory studies. Here we present all genomic changes, from single nucleotide polymorphisms (SNPs) to large structural variants, between isolate pairs in order to gain insights into the bacterial genetic adaptations during persistent infection.

## Results

### Experimental Approach

To comprehensively investigate genetic changes in *B. pseudomallei* during persistent infections, we analyzed genomic data for clonal primary-relapse isolates collected from four patients admitted to Sapprasithiprasong Hospital in Thailand between 1988 and 1999 (Table 1). Clonality was initially established with multilocus sequence typing (MLST) [19]. The intervening periods for these pairs spanned from six months to more than six years, a range that overlaps that of a majority of reported relapse episodes [10]. We also analyzed genomic data for a pair of clonal primary isolates collected from one patient in 1993 (Table 1). These isolates were sampled from different body sites on the day of hospital admittance and were included in the study to provide insight into genetic changes during acute primary infection.

**Table 1:** *B. pseudomallei* isolates used for whole genome comparison.

Clinical features+											
Isolate	Year	Time between isolates (mo)	ST*	Specimen location	Age (yr)	Sex	Symptom duration# (dy)	Diabetes	Other risk factor	Organs involved	Blood culture
1026a	1993		102	Pus	29	F	14	Yes	No	Skin, soft tissue, spleen, joint	Pos
1026b	1993	0	102	Blood							
1258a	1994		221	Sputum	72	M	10	Yes	No	Lung	Neg
1258b	1995	6	221	Blood							
1710a	1996		177	Blood	52	M	7	Yes	No	Lung, soft tissue abscess	Pos
1710b	1999	30	177	Blood							
1106a	1993		70	Liver pus	21	F	3	No	Thalassaemia	Liver	Neg
1106b	1996	36	70	Liver pus							
354a	1988		78	Sputum	65	M	9	No	No	Lung	Neg
354e	1994	75	78	Sputum							

* Multilocus sequence types (ST) categorize strains based on variations in DNA sequence of seven housekeeping genes [19].

+ Clinical details are provided for the primary episode for each patient.

# Days of symptoms prior to presentation at the hospital.

We aligned raw sequence reads to reference genomes to assess SNPs and insertions/deletions (indels) within clonal pairs. Publicly available completed genomes for 1106a and 1710b and Sanger sequence reads for 1106b and 1710a provided for direct comparison of primary and relapse isolates in the 1106 and 1710 pairs. We generated sequence reads for 1026a (GenBank accession AHJA00000000) using Illumina sequencing-by-synthesis technology and aligned those reads to our completed 1026b genome. We also generated Illumina reads for 1258a (GenBank accession AHJB00000000), 1258b (GenBank accession AHJC00000000), 354a (GenBank accession AGVS00000000) and 354e (GenBank accession AHJD00000000), and we identified variants unique to the primary or relapse isolate in these pairs based on alignment of reads to the 1026b and K96243 [20] reference sequences. All SNPs and short indels were validated by PCR and Sanger sequencing.

To assess structural variation within clonal pairs, we employed a fosmid cloning strategy designed to identify large-scale (>1 Kb) differences between a query and reference genome [21], [22]. A set of fosmid clones for each isolate listed in Table 1 was aligned, or tiled, against the appropriate reference sequence to reveal insertions, deletions, inversions and translocations. Self-tilings, such as 1026b fosmids tiled against the 1026b reference sequence, served as controls, and in this particular example, also aided with validation of the completed 1026b sequence.

### Genome Sequence of the *B. pseudomallei* 1026b Reference Strain

**Figure 1:**
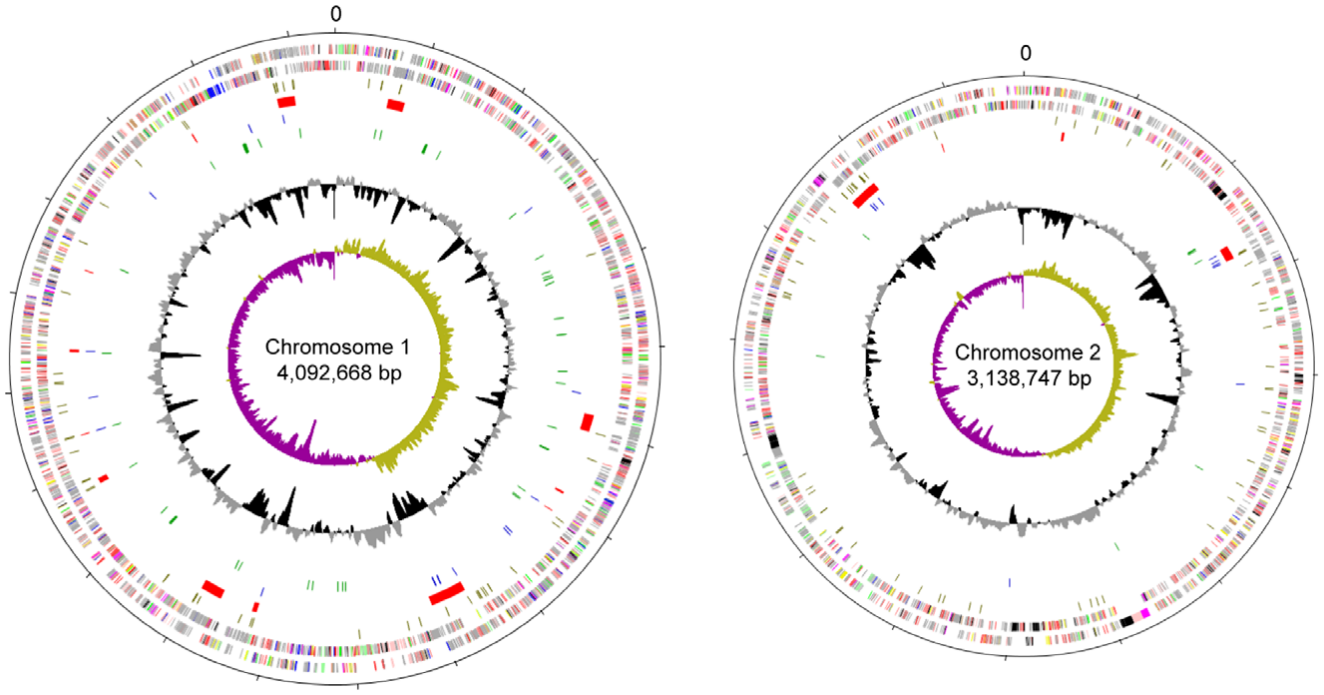
Schematic circular diagrams of the large and small chromosomes of the 1026b genome. Rings from outside to inside: scale with tick marks positioned every 200 Kb, annotated forward CDS and reverse CDS colored-coded by COG functional categories (Table S1), pseudogenes (olive), genomic islands (red), IS elements (blue), rRNA and tRNA (green), GC% plot and (G – C)/(G + C) deviation plot.

*B. pseudomallei* 1026b was isolated from a 29-year-old female patient with septicemic melioidosis with skin, soft tissue, spleen and joint involvement (Table 1). The strain has become a model for genetic analysis of *B. pseudomallei* due to its high efficiency of transformation and overall ease of genetic manipulation [23], [24]. An attenuated strain suitable for biosafety level 2 (BSL2) manipulation also has been constructed [25]. Strain 1026b is the fifth *B. pseudomallei* clinical isolate to be sequenced to base-pair accuracy (www.ncbi.nlm.nih.gov/). We annotated the 1026b sequence and compared it to four completed and nine draft *B. pseudomallei* genomes using the Prokaryotic Genome Analysis Tool (http://tools.nwrce.org/pgat/) [26]. We found that 1026b was similar to the others with respect to genome size and structure, number of predicted genes and other attributes (Figure 1, Table S1 and Table S2). Of the 6070 total predicted genes in 1026b, 4705 (77%) were present in all 13 of the other genomes, 1330 (22%) were shared by a subset, and 35 (1%) coding primarily phage and hypothetical proteins were unique to 1026b (Table S3). The number of core genes is comparable to the 4908 identified by Nandi et al. [18] for a partially overlapping set of 11 genomes, and the difference is likely due to the larger number of draft quality genomes in our data set. When compared to just the four other completed *B. pseudomallei* genomes (Table S2), there were 31 genes that were present in those strains but were missing or interrupted (pseudogenes) in 1026b (Table S3). In addition to hypothetical proteins, this list includes the entire K96243 genomic island (GI) 1 and the gene encoding the signal transduction histidine kinase IrlS2.

We identified 17 GIs in the 1026b genome (Table 2), including the functional prophage phi1026b [27]. Ten GIs are shared at least in part by one or more of the other four completed genomes, including five islands that were described in K96243 [20]. Two additional K96243 islands (GI7 and GI14) that were present in all 94 *B. pseudomallei* strains evaluated by Sim et al. using a K96243-based DNA microarray [28] are present in the 1026b genome and are considered to be part of the core genome, following their suggestion. Of the seven islands not found in other completed genomes, two contain genes for XRE family transcriptional regulator proteins, one contains a putative ATP binding protein gene and four contain genes encoding prophage and/or hypothetical proteins. In addition to the GIs identified in our study, 1026b contains 16 of the 20 novel indels identified by Sim et al., where novel was defined as the absence of at least three contiguous probes in at least three of their 94 strains [28]. The 1026b genome is missing all genes in indels 1 and 2, which encode hypothetical and phage proteins. It also is missing most genes in indels 4 and 11, with the exception of a homolog of an exported protein (gene locus BPSL2038 in K96243) and a pseudogene of pANL56 (BPSS0395) from those two indels, respectively.

The characteristic composition and organization of this genome substantiates the use of 1026b as the representative *B. pseudomallei* isolate for laboratory studies.

**Table 2:** Genomic islands in *B. pseudomallei* 1026b.

Island	Locus tags	Size (kb)	Mobility-related genes*	GC (%)	Genes in K96243 genomic islands (shared/total)	Presence in other completed genomes+	Functional note
1	BP1026B_I0126-BP1026B_I0181	40.5	1 Int	64.8	GI2 42/48 GI15 28/43	+	Prophage
2	BP1026B_I1081-BP1026B_I1137	42.7	1 Int	63.5	–	–	Prophage
3	BP1026B_I1268-BP1026B_I1271	4.7	1 Int	61.0	–	–	Hypothetical proteins
4	BP1026B_I1319-BP1026B_I1320	1.1	2 Tnp	61.1	–	+	Transposases
5	BP1026B_I1584-BP1026B_I1662	90.8	3(1) Tnp	62.0	GI8 55/57	+	Transport proteins, outer membrane proteins and transcriptional regulators
6	BP1026B_I2008-BP1026B_I2017	10.4	3 Tnp	53.1	–	+	Hypothetical proteins
7	BP1026B_I2085-BP1026B_I2161	54.5	1 Int	60.8	–	+	phi1026b
8	BP1026B_I2478-BP1026B_I2482	12.5	0	60.6	–	+	Hemolysin activator protein precursor and haemagluttinin repeat protein
9	BP1026B_I2589-BP1026B_I2592	1.5	1 Tnp	60.0	GI5 3/9	+	Putative plasmid replication protein
10	BP1026B_I2779-BP1026B_I2784	8.2	1 Int, 2 Tnp	49.8	–	–	XRE family transcriptional regulator and plasmid recombination enzyme
11	BP1026B_I2942-BP1026B_I2943	4.5	0	55.0	–	–	Putative ATP binding protein and hypothetical protein
12	BP1026B_I3340-BP1026B_I3346	4.7	0	59.6	–	–	Prophage proteins
13	BP1026B_I3580-BP1026B_I3637	43.4	1 Int, 1 Tnp	63.6	GI2 34/48 GI15 26/43	+	Prophage
14	BP1026B_II0077-BP1026B_II0080	5.8	1 Tnp	55.1	–	+	Putative ABC transport proteins
15	BP1026B_II0420-BP1026B_II0449	28.8	2 Int, 7(2) Tnp	57.5	–	–	XRE family transcriptional regulator and hypothetical proteins
16	BP1026B_II2203-BP1026B_II2252	62.1	2 Int, 6(1) Tnp	59.2	GI16 21/37	+	Metabolic island
17	BP1026B_II2392-BP1026B_II2393	1.1	0	50.1	–	–	Hypothetical proteins

* Integrase and transposase are abbreviated as Int and Tnp, respectively. Partial genes and pseudogenes are indicated in parentheses.

+ Other completed genomes are 1710b, 1106a and 668.

### Comparison of 1026a and 1026b Primary Isolates

The 1026a and 1026b isolates were collected from pus and blood, respectively, 14 days after the onset of symptoms (Table 1). Based on the data we collected, the genomes of these two primary isolates were identical (Table 3).

**Table 3:** Summary of whole genome variation in the five *B. pseudomallei* isolate pairs.

Isolate pairs	# SNPs	# Indels	Structural variants	Chromosome
1026a-1026b	0	0	None	-
1258a-1258b	0	0	330 Kb Deletion	2*
1710a-1710b	2	14	22 Kb Tandem repeat	2*
1106a-1106b	5	41	None	-
354a-354e	8	2	800 Kb Inversion	2*

* 1258a, 1710a and 354e exhibited the structural variant noted.

Three previous studies based on more limited data investigated genetic changes among *B. pseudomallei* isolates during acute infections. In their study of four acute Thai cases, Price et al. reported that roughly 70% of isolates were identical to that of founder genotypes at 23 variable number tandem repeat (VNTR) loci, while 30% were observed to have on average one or two repeat copy number changes [17]. These VNTR regions span simple sequence repeats (SSRs), which are tandem arrays of short sequences that gain or lose repeat units by recombination or slipped-strand mispairing during DNA replication [29]. The *B. pseudomallei* genome contains a large number of SSRs relative to other bacterial pathogens of similar genome size and GC content [30], [31], and Price et al. demonstrated that the *in vitro* mutation rate of these sites in *B. pseudomallei* is high relative to SSRs in *Yersinia pestis* and *E. coli* [17]. Pearson et al. found a similar amount of VNTR variation among isolates collected from an Australian patient with fatal septicemic melioidosis [15]. Sam et al. studied five *B. pseudomallei* isolates sampled from a single Malayan patient at days 1 (3 isolates), 9 (1 isolate) and 17 (1 isolate) of admission, during which he was treated with ceftazidime [16]. They identified a single amino acid change in the class A β-lactamase gene *penA* in two isolates from day 1 and one isolate from day 17 that conferred increased resistance to ceftazidime and increased susceptibility to amoxicillin-clavulanate.

These studies suggest that genetic changes occur in subpopulations of *B. pseudomallei* during acute infections; thus, the lack of variation observed between 1026a and 1026b may be the result of limited sampling that fails to detect changes, especially rare events, in the infection. Certain changes, particularly those at VNTR loci, may be unrelated to bacterial fitness, while others, such as *penA* mutations, are adaptive in response to treatment regimes. This pattern of change for acute primary disease provided a basis for understanding the mutational process in recurrent melioidosis.

### Indels are the Most Abundant Form of Genomic Variation Among Primary-relapse Pairs

We found 57 indels among three of the four primary-relapse pairs (Table 3); 33 were intergenic and 24 were located in coding sequence (Table S4). No indels were found in 1258a-1258b, the pair with the shortest time interval between isolates. All indels, regardless of being located in coding or intergenic sequence, occurred in SSRs. Pairwise comparisons revealed one recurrent variant in the 1106 and 1710 pairs that did not change the amino acid sequence of a hypothetical protein (Table S4). In the 1106 and 1710 pairs, indels in coding regions were almost evenly split between chromosome 1 and chromosome 2, and between insertions and deletions, while in 354a-354e they were restricted to deletions in chromosome 2 (Table S4).

In-frame (3 bp triplet) insertions and deletions altered the number of amino acid repeats in the relapse isolate relative to the primary isolate. Indels involving non-triplet repeat motifs had various consequences. Frameshifts occurring within the first quarter to third of the protein, which presumably disrupt protein function, were most abundant. In the 1106 and 1710 pairs, all predicted frameshifts in the relapse isolate occurred in proteins annotated solely as hypothetical, except frameshifts in two known genes that may be the result of start codon misassignment (Table S4). In the 354 pair, frameshifts occurred near the start of a short-chain dehydrogenase gene locate immediately downstream of *folE* involved in folate biosynthesis, and in the gene encoding lipase-like protein LlpE that is located between the BpeEF-OprC drug efflux system and its LysR type regulator BpeT. In addition to frameshifts, we found insertions in 1106b near the end of open reading frames that resulted in: 1) no apparent changes to the amino acid sequence, 2) non-synonymous substitutions with no alteration in the length of the protein, or 3) non-synonymous substitutions resulting in removal of the stop codon. In the last case, the protein, annotated as an AraC family transcriptional regulator, is extended by 94 amino acids.

Given the very high SSR mutation rate in *B. pseudomallei* [15], [17], [30] and the preponderance of changes in intergenic regions, much of the observed variation is likely to be selectively neutral. In coding regions the pattern of SSR variation resulting in changes in amino acid repeat units has been observed previously in *B. pseudomallei* and has been implicated in phase variation, the process by which genes undergo reversible mutations and generate phenotypic diversity in clonal populations [31], [32]. Interestingly, variable copy numbers of the in-frame repeat units listed in Table S4 were found in the same genes in unrelated *B. pseudomallei* strains with publicly available sequences (not shown); however, the functional significance of these changes remains unknown. Further experiments are also required to understand the phenotypic consequences of observed frameshifts in coding regions. The frameshift in LlpE is particularly intriguing, since we also found a structural variant within the BpeEF-OprC operon of 354e as described below.

### Point Mutations Indicate Variation within Single Infections of *B. pseudomallei* in Two Melioidosis Patients

Despite varying time intervals between primary and relapse isolates (Table 1) and different methods of SNP detection (see Materials and Methods), the number of point mutations within clonal pairs ranged from zero to eight. These results are consistent with the observed number of SNPs in the 1710 and 1106 pairs made by Nandi et al. [18], although their list contains errors in the 1710b and 1106a reference sequences (consensus bases not supported by Sanger or Illumina reads, Table S5) that could not be identified as such by their methods. Of the fifteen SNPs identified in the 1710, 1106 and 354 pairs, thirteen occurred in coding sequence (Table 4). Eight resulted in nonsynonymous (NS) changes, and seven of these occurred in genes with known or putative functions. Five NS changes were predicted to be deleterious by the “Sorting Intolerant from Tolerant” (SIFT) algorithm, which computationally predicts whether an amino acid substitution will be deleterious (D) or tolerated (T) for protein function [33]. Of particular note were two mutations that were present in primary isolates but absent from the associated relapse strains. The synonymous mutation in the CobN gene of 354a was unique relative to 354e and all other *Burkholderia* strains in BLASTN alignments. Similarly, the NS mutation in the TetR family transcriptional regulator protein of 1710a was unique relative to 1710b and all other *Burkholderia* strains in BLASTP alignments. While it is possible that these SNPs were not found in relapse isolates due to reversion of nucleotide changes, a more parsimonious explanation is that the primary and relapse isolates were sampled from divergent lineages of the bacterial infection. Variation in single melioidosis infections has been observed previously [15], [17]. As discussed below, we found more evidence for lineage heterogeneity from additional SNP discovery and analysis of structural variants.

**Table 4:** SNPs found in the 354, 1106 and 1710 isolate pairs.

Reference locus	Isolate	S/NS	AA	AA in *Burkholderia* spp.	AA/Total	SIFT#	Gene product	Protein function
BPSL0042	354e	NS	Cys	Arg	361/670	T	Type III DNA modification methyltransferase	Defense mechanism
BPSL1768	354a	S	-		1107/1281	-	Cobaltochelatase (CobN)	Vitamin B12 metabolism
BPSL2301	1710b	S	-		35/898	-	Pyruvate dehydrogenase subunit E1 (AceE)	Energy production
BPSL2706	354e	NS	Lys	Thr	271/532	D	Putative lipoprotein	-
BPSL3027	1106b	S	-		142/504	-	DP-N-acetylmuramoyl-L-alanyl-D-glutamate synthase (MurD)	Cell envelope biogenesis
BPSL3288	354e	NS	Leu	Pro	216/276	D	5,10-methylenetetrahydrofolate reductase (MetF)	Methionine metabolism
BPSL3389	1106b	NS	Val	Ala	886/1309	D	Trifunctional transcriptional regulator/proline dehydrogenase (PutA)	Energy production
BPSL3390	1106b	S	-		645/780	-	Primosome assembly protein (PriA)	DNA replication
BPSS0289*	354e	-	-	-	-	-	Hypothetical protein	-
BPSS0571	1106b	S	-		76/117	-	Hypothetical protein	-
BPSS0946+	354e	-	-	-	-	-	β-lactamase (PenA)	Defense mechanism
BURPS1106A_1241	1106b	NS	His	Arg	45/178	-	Hypothetical protein	-
BPSS1483	354e	NS	Phe	Leu	41/344	D	TetR family protein	Transcriptional regulation
BPSS1483	1710a	NS	Asp	Tyr	53/344	D	TetR family protein	Transcriptional regulation
BPSS1995	354e	NS	Thr	Ala	58/475	T	Signal transduction histidine kinase (IrlS2)	Signal transduction

* SNP is located 112 bp upstream of BPSS0289.

+ SNP is located 78 bp upstream of penA.

# SNPs are predicted to be deleterious (D) or tolerated (T) with regard to protein function.

### Mutations in a TetR Family Transcriptional Regulator Reveal Adaptation of *B. pseudomallei* in Clinical Isolates

Pairwise comparison of all SNPs showed recurrent mutations in homologs of the TetR family transcriptional regulator BPSS1483 in 1710a and 354e (Table 4). Mutations were identified at different positions, but in both isolates the SNP occurred early in the protein at highly conserved residues [34]. TetR family proteins are transcriptional repressors that control genes whose products are involved in multidrug resistance, biosynthesis of antibiotics, osmotic stress, pathogenicity and various catabolic pathways in gram-negative and gram-positive bacteria. The discovery of two independent NS mutations in the same TetR family regulator prompted us to investigate the sequence of this gene in additional *B. pseudomallei* isolates. We screened for mutations in homologs of BPSS1483 in 107 strains from Thailand, Australia, Pakistan, Singapore and Vietnam (Table S6). This list included 32 clonal primary-relapse pairs and eight environmental strains. We found 22 sites with NS mutations in 18 clinical isolates. Nearly half of these mutations occurred in primary-relapse pairs; none were found in any of the environmental strains (Table 5). We found eight sites with synonymous changes in a total of nine isolates (Table S7).

**Table 5:** Nonsynonymous mutations in BPSS1483 homologs from 107 *B. pseudomallei* isolates.

Nucleotide change	Amino acid change	Strain	Present in primary	Present in relapse+	Predicted effect on protein function#
C70T	Q24Stop	770	N	Y	Stop
C160T	R54C	242, 1350*	N, N	Y, Y	D
T185G	L62R	1428*	N	Y	D
G307T	E103Stop	1686*	N	Y	Stop
G351A	W117Stop	479	N	Y	Stop
G358T	E120Stop	479	Y	N	Stop
T500C	L167P	Pakistan 9	Y	-	D
G539A	W180Stop	986a	Y	-	Stop
C647T	A216V	668	Y	-	-
C686T	A229V	1655	Y	-	-
C691G	Q231E	1119, 1210, 1069c	Y, Y, Y	Y, Y, -	-
G695A, C696T	G232D	1119, 1210, 1069c	Y, Y, Y	Y, Y, -	-
G703C	E235Q	668, 305	Y	-	-
A709G	T237A	668	Y	-	-
G712A	A238T	305, 1069c	Y, Y	-, -	-
G838A	G280S	668, 406e, Pasteur 52237, 1373c	Y, Y, Y, Y	-, -, -, -	-
A893G, T894C	D297G	1069c	Y	-	-
C904T	P302S	658	Y	Y	-
C973T	P325S	668, 305	Y, Y	-, -	-
T979A	S327T	305	Y	-	-
C982T	S328P	305	Y	-	-
A988G	T330G	305	Y	-	-

* Sequence chromatograms showed peaks for both the reference and SNP bases at this position in relapse isolates.

+ For isogenic pairs only.

# SNPs introduced a stop codon (Stop) or were predicted to be deleterious (D) with regard to protein function. Prediction was not possible in the second half of the gene due to gaps in sequence alignments.

Similar to most prokaryotic transcriptional regulators, TetR family proteins possess helix-turn-helix (HTH) DNA-binding motifs, which in TetR proteins are located in the N-terminal region and show a high degree of sequence conservation among family members [34]. All NS mutations found in the first half of the gene introduced either a stop codon or an amino acid that was predicted to be deleterious to protein function (Table 5). In addition, we found a single base pair deletion at position 448 in a primary isolate with no associated relapse that caused a frameshift in the coding sequence (not shown). The second half of the gene contains SSRs and numerous strains had trinucleotide-based indels relative to the K96243 reference sequence, which introduced alignment gaps. As a result, predictions of amino acid changes on protein function were not possible here. Nonetheless, it is interesting that most of these mutations were found in strains from Australia, e.g. 668, 305 and 1655. Australian and Asian strains have been shown to be genetically distinct [18], [35]; thus, these SNPs may simply reflect geographical differences. It is also interesting that NS changes here are shared by primary and relapse isolates of clonal pairs, e.g. 1119, 1210 and 658. This is in contrast to the deleterious changes in the first half of the gene, most of which were found in only the relapse isolate of a given pair.

For comparison, we searched for NS SNPs in 20 additional genes annotated as TetR family regulators in 20 publicly available *B. pseudomallei* genomes, including one environmental strain. We found ten genes with ≤1 NS SNP, eight with 3–6 NS SNPs and two with >6 (10 and 17) NS changes (not shown). In this set of genomes there were 10 NS changes in BPSS1483. The environmental strain, *B. pseudomallei* S13, had single NS SNPs in two regulators, both of which occurred within the final ten amino acids of the proteins, were shared with at least two other strains and were predicted to be tolerated or were in a region where predictions were not possible. Taken together, it appears that BPSS1483 homologs experience a relatively high rate of NS substitutions compared to most other TetR transcriptional regulators.

The 5′ end of BPSS1483 is likely crucial for function, as suggested by the high level of sequence conservation among TetR family members and the presence of identifiable domains. While synonymous and non-synonymous substitutions were evenly distributed across the gene, the conserved 5′ region contained several nonsense and deleterious mutations specific to relapse clinical isolates. Although the data set was not amenable to dN/dS calculation, the density of deleterious changes in this region and this group of strains, combined with the high rate of NS substitutions in BPSS1483 homologs relative to other TetR transcriptional regulators, lead us to speculate that there is positive selection for coding changes in this gene and that it may play an adaptive role *in vivo*.

### BPSS1483 Homologs are Present in Other Pathogens but the Gene does not Affect Antibiotic Susceptibility

Little is known about BPSS1483 beyond being annotated as encoding a TetR family protein. A search for the gene in the STRING database [36] showed that synteny of BPSS1483 with the adjacent and divergently oriented hypothetical proteins BPSS1484, BPSS1485 and BPSS1486 is preserved in strains of *B. pseudomallei* and its close relatives *B. mallei* and *B. thailandensis*. Syntenic homologs of these four genes also were found in the pathogens *Bordetella pertussis*, *B. parapertussis* and *B. bronchiseptica*, and syntenic homologs of BPSS1483, BPSS1484 and BPSS1485 were identified in the human pathogens *Yersinia pestis* and *Y. enterocolitica*, and several plant and insect pathogens or endosymbionts, including *Erwinia carotovora*, *Photorhabdus luminescens* and *Rhizobium leguminosarum*. The *Bordetella* and *E. carotovora* genomes possess 15 or more TetR proteins, while the *Y. pestis* and *P. luminescens* genomes contain only 6–8 [34]. The presence of this putative BPSS1483 operon in numerous pathogens and symbionts, especially among the relatively small number of *tet*R-related genes in *Y. pestis* and *P. luminescens* genomes, supports the hypothesis that these genes contribute to the ability of bacteria to survive *in vivo*.

Since TetR regulators are sometimes involved in antibiotic resistance, we tested the susceptibility of three *B. pseudomallei* and three *B. thailandensis* strains to ten antimicrobial compounds (Table 6). The *B. pseudomallei* strains included 1026b, 354b and 354e. The 354b isolate was collected during the primary infection three months after the 354a isolate, which could not be recovered in culture. The sequence of the BPSS1483 homolog from 354b was identical to that of 354a. The *B. thailandensis* strains included the sequenced reference strain E264 and two transposon insertion mutants from a *B. thailandensis* E264 sequence-defined transposon mutant library (L. Gallagher and B. Ramage, pers. comm.). TnBTH_II0885 has a transposon insertion in the BPSS1483 homolog. TnBTH_I3273 has an insertion in a transposase gene and was included as an insertion mutant control (see Methods and Materials). *B. thailandensis* has been used as a surrogate for studying many different traits in *B. pseudomallei* [37], [38], since it can be manipulated under BSL2 laboratory conditions and has no restrictions on use of antibiotic resistance markers for research. These mutants carry a tetracycline resistance gene and were expected to have high MICs for this compound.

**Table 6:** Antibiotic susceptibility of *B. pseudomallei* and *B. thailandensis* strains.

	MIC (ug/ml)									
	TZ	CL	IP	MC	MP	OF	PP	TC	TS	XL
***B. pseudomallei*** ** strains**										
1026b	1.5	8	0.38	0.75	1.5	2	1	1.5	0.25	1
354b	2	12	0.5	1.5	1.5	3	2	2	0.38	2
354e	6	>256*	1.5	6	6	>32*	4	4	3	4
***B. thailandensis*** ** strains**										
E264	1.5	8	0.38	1	1	1.5	2	3	0.75	3
TnBTH_I3273	1.5	8	0.38	4	1	3	2	64	1.5	3
TnBTH_II0885	1.5	8	0.38	2	1	1.5	2	64	1.5	6

Antimicrobial agent abbreviations: TZ, ceftazidime; CL, chloramphenicol; IP, imipenem; MC, minocycline; MP, meropenem; OF, ofloxacin; PP, piperacillin; TC, tetracycline; TS, trimethoprim/sulfamethoxazole (1/19); and XL, amoxicillin/clavulanic acid (2/1). For TS and XL, numbers in parentheses indicate the ratio of drugs in the combination, and the MIC value refers to the first component.

* Detection limits of the assays.

The most striking result of the susceptibility test was the high MICs displayed by 354e relative to the other strains. In particular, resistance increased >21-fold and >10-fold to chloramphenicol and ofloxacin, respectively, and nearly 8-fold to trimethoprim/sulfamethoxazole compared to the primary isolate. The TnBTH_II0885 mutant did not exhibit a similar resistance phenotype, suggesting that something other than the BPSS1483 mutation in 354e accounted for reduced susceptibility to these antimicrobials. As discussed below, 354e has an inversion in chromosome 2 that interrupts the BpeT regulator of the BpeEF-OprC drug efflux operon, which has been shown to efflux chloramphenicol and trimethoprim [39]. Additionally, we found a SNP in 354e located 78 bp upstream of the β-lactamase gene *penA* (Table 4) known to mediate β-lactam resistance in *B. pseudomallei* [40], which may play a role in its reduced susceptibility to those compounds in our panel.

### Structural Variants Provide Further Evidence for Lineage Heterogeneity and Adaptation During Infection

In three of the four primary-relapse pairs, we found large structural variants of chromosome 2 that interrupted otherwise syntenic primary and relapse replicons (Table 3). In the 354 pair we found an 800 Kb inversion in the relapse isolate relative to the primary isolate and other complete *B. pseudomallei* genomes (not shown). The inverted segment of DNA contains the origin of replication. One breakpoint is located in a transposase gene homologous to BPSS2046, and the other is located in the C-terminal region of the homolog of BpeT, the regulator of the BpeEF-OprC drug efflux system. This rearrangement of chromosome 2 may contribute to reduced susceptibility of 354e to chloramphenicol and trimethoprim as discussed above. Inversions of this size have been observed in chromosome 1 of several *B. pseudomallei* strains, including K96243, 1655, Pasteur52237 and 406e [18]; however, this is the first report of such an inversion in chromosome 2.

**Figure 2:**
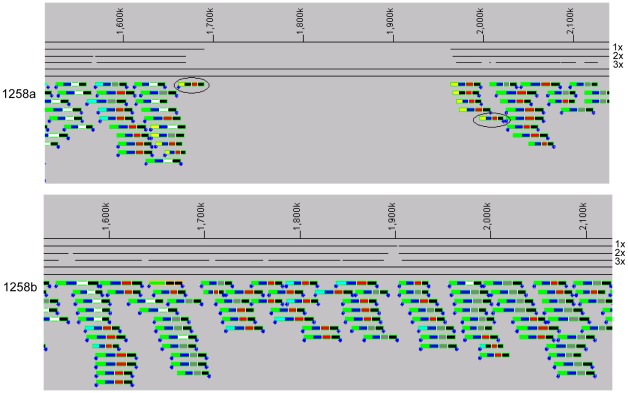
Fosmid tilings from 1258a and 1258b spanning the location of the 330 Kb deletion. Tilings of 1258a (top) and 1258b (bottom) fosmids against the 1026b reference genome as shown. Sequence coordinates of 1026b chromosome 2 appear along the top horizontal line below which three black lines indicate 1x, 2x and 3x fosmid coverage. Two additional horizontal black lines visually separate coverage lines from clone positions, which are represented by rectangles. Four colors within each rectangle illustrate features of the clone position as described in Hayden et al. [21]. Blue dots at the lower left and right edges of rectangles symbolize the x and y fosmid paired-end sequences aligned to the 1026b sequence. In 1258a, five clones (yellow first quarter) spanned the deletion breakpoint and flanked a large coverage gap in the tiling. The x (at 2.0 Mb) and y (at 1.7 Mb) anchored positions of a single clone are circled. In contrast, clones from 1258b tiled throughout this region indicating that there is no deletion in this isolate.

In the 1258 pair we found a 330 Kb deletion in chromosome 2 of the primary isolate relative to the relapse isolate and other complete *B. pseudomallei* genomes (Figure 2). This is the only genomic variation that we found in this isolate pair, i.e. the primary and relapse genomes are otherwise identical based on the data we collected. One end of this large deletion is located intergenically between homologs for a putative lipoprotein (BPSS1249) and acetylpolyamine aminohydrolase (BPSS1250) and the other is located within BPSS1483 resulting in the deletion of nearly 40% of this *tetR*-related gene. This deletion removed 232 complete genes (homologs of BPSS1250-BPSS1482) from chromosome 2 of 1258a totaling 10% of the replicon. The deleted genes are part of a highly syntenic region of the core *B. pseudomallei* genome supporting the notion that DNA was lost from 1258a rather than gained by 1258b. Given that 1258a and 1258b are clearly clonal, a reasonable scenario is that the patient contained at least two lineages, one with and one without the deletion, at the time the primary isolate was collected. During relapse, only the isolate without the deletion remained, or was sampled. Accordingly, we believe that this deletion is another example of genetic heterogeneity within a single infection of *B. pseudomallei*.

**Figure 3:**
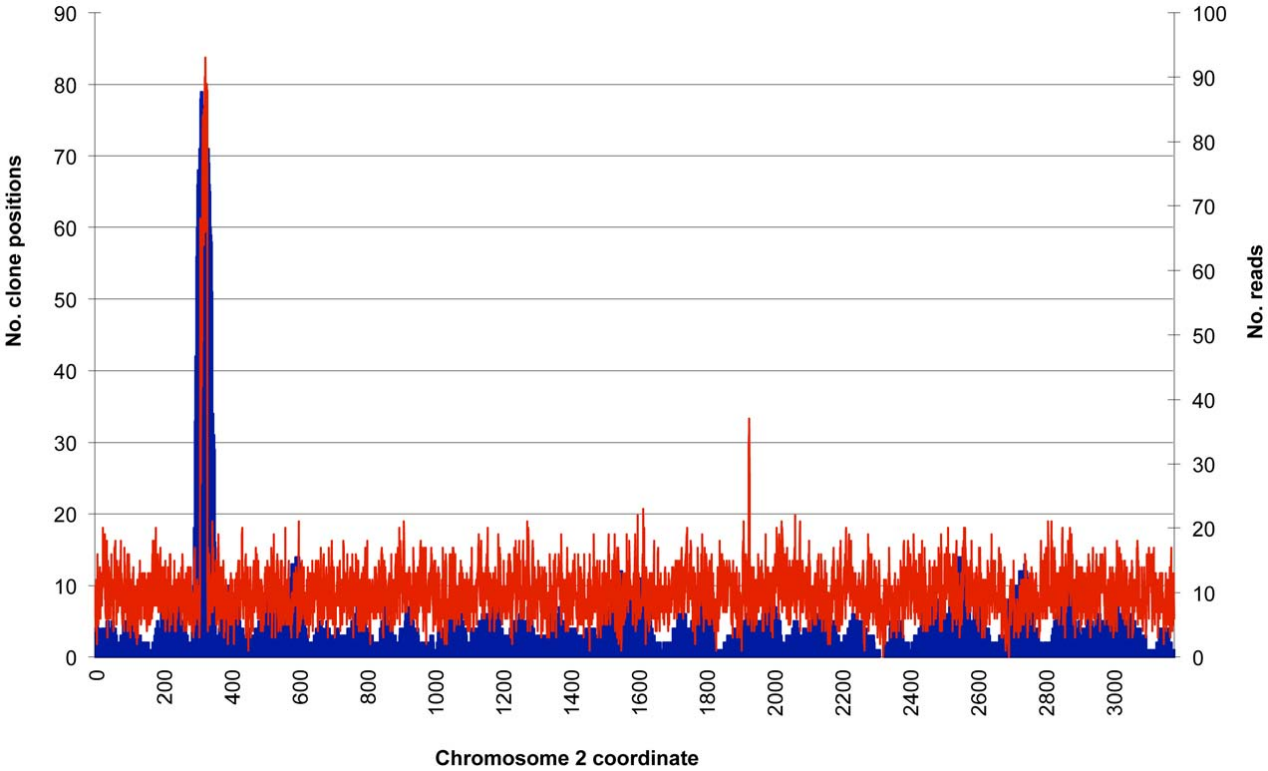
Depth of coverage of 1710a Sanger sequence reads and fosmids across chromosome 2. The number of 1710a sequence reads (red) and fosmids (blue) was counted in 500 bp and 10 Kb sliding windows, respectively, along chromosome 2 of the complete 1710b reference sequence. The spike in coverage of fosmids and sequence reads between 200 and 400 Kb is due to the tandem repeat of 17 genes. The smaller spike of sequence reads between 1800 and 2000 Kb is the result of a repeat of the Rhs element Vgr protein gene BURPS1710b_A0207.

The most intriguing structural variant identified in our study was found in the primary isolate of the 1710 pair relative to the relapse isolate and other completed *B. pseudomallei* genomes. In this isolate we found a tandem repeat comprised of a 22 Kb region of chromosome 2. The depth of coverage of 1710a Sanger sequence reads and fosmids aligned to the 1710b reference sequence in this region was approximately 7 and 12 times the average, respectively (Figure 3). We estimated that there were likely closer to 7 copies in the repeat based on limitations of fosmid tiling; however, the exact copy number in the repeat was not elucidated, since the repeat size confounded sequence finishing. The repeat spanned 17 genes, extending from a gene encoding a rearrangement hot spot (Rhs) element Vgr protein to a gene encoding a terpene synthase family protein (Table S8). Among the 17 genes was a penicillin-binding protein (PBP) (BPSS1219) whose deletion has been associated with resistance to ceftazidime [41]. Amplifications of PBP genes also have been shown to increase β-lactam resistance [42], e.g. overproduction of the related PBP3 in *Pseudomonas aeruginosa* has been linked to reduced susceptibility to this class of compounds [43]. Thus, it is reasonable to hypothesize that amplification of this gene in 1710a was a result of antibiotic selective pressure, as ceftazidime has been used as first line parenteral therapy in Thailand since 1989 [44].

## Discussion

In this whole-genome study of sequential *B. pseudomallei* clinical isolates, we described the extent of genetic variation from SNPs and indels to large-scale structural changes. There were notable patterns among the pairs. We discovered nucleotide changes and structural variants in primary isolates that were absent from associated relapse isolates and identified genomic changes in both primary and relapse isolates that likely increased resistance to antimicrobials administered for treatment. We also identified recurrent deleterious mutations in a TetR family transcriptional regulator that may promote survival *in vivo*. It is unlikely that these observed variations were due to reinfection of patients with strains of different genotypes. The paired isolates are unambiguously clonal, and the chance is very low of infection months or years later by the same clone given the vast diversity of strains found in the environment [45]. It is likely that some variation existed within the population of cells that comprised the early stages of infection. Given observed mutation rates of VNTR loci [17], [30], some diversity in SSR lengths could be expected, especially for repeats in non-coding regions. However, outside SSRs, it is most likely that the observed changes were selected for in the host. The strongest evidence for this comes from variation in the TetR family repressor, which showed evidence of negative selection for its function in relapse clinical isolates despite conservation of homologs across an array of plant and animal pathogens. Further evidence comes from observed variations in or near genes involved in antibiotic resistance mechanisms, such as multidrug efflux pumps, penicillin-binding proteins and β-lactamases. Taken together, our results reveal the presence of multiple genotypes within a single infection that are, at least in part, the result of genetic adaptation to the human host.

Among our four primary-relapse pairs, we did not detect the loss of virulence factors as has been reported in studies of sequential isolates from chronic infections of *Pseudomonas aeruginosa* [2], [4]. We speculate that bacteria that cause a relapse episode after the patient is apparently cured may be responding to a different set of selection pressures than those involved in chronic disease. Although little is known about the long-term persistence of *B. pseudomallei in vivo*, the bacterium has been shown to quiescently persist *in vitro*. Pumpuang et al. recently demonstrated survival of *B. pseudomallei* in distilled water for 16 years [46]. They found that the number of live organisms estimated by viability staining was higher than that seen from a colony count on agar, which suggested a proportion of the surviving cells were non-culturable under standard conditions, perhaps due to being in a non-replicating state. Also recently, Hamad et al. showed that under anaerobic conditions, a small proportion of the *B. pseudomallei* population maintained complete viability for at least one year in an apparently dormant state and was sensitive to drugs used against anaerobic pathogens but not to traditional melioidosis antibacterials [47]. They likened this persister population to those found in other bacteria, such as *Mycobacterium tuberculosis* that can cause recurrent infections due to relapse. Incidentally, analysis of IS6110 DNA patterns in clonal *M. tuberculosis* strains separated by 30 years showed stability compared to patterns in active disease, demonstrating that these persistent bacteria were not actively replicating [48]. The existence of a non- or slow-replicating state during persistent infections may be key to the types of genomic changes observed.

The amount of genetic variation observed across the four primary-relapse pairs was relatively consistent, and the number of SNPs increased as time between primary and relapse isolates increased (Table 3). With the exception of SNPs in BPSS1483 homologs and one recurrent indel in a hypothetical protein, there was no overlap in mutations across isolates. This may be explained by three non-mutually exclusive possibilities: 1) we did not find all the existing variation in our isolate pairs, 2) our sample size was too small, especially for detecting rare variants, and 3) the human host influences evolution of the pathogen. Detection of indels in SSRs longer than the length produced by current short read sequencing technologies is difficult. Similarly, detecting SNPs in repeat regions can be difficult due to poor sequence read mapping. Our fosmid-based method for identifying structural variation has size limitations as well [21]. Despite these shortcomings, it is reasonable to believe that we found most of the existing variation given our mix of sequence data types and analysis methods and the fact that our results are consistent with a previous study [18]. Recurrent mutations in genes such as *penA* documented in multiple non-clonal strains elsewhere [16], [49] were not recurrent in our dataset. Our sampling allowed us to uncover a portion of the genetic variation in four independent infections. We recognize that more than two isolates are required to fully characterize the evolution of *B. pseudomallei* over the course of a single infection and anticipate that a study sampling several isolates at each time point would uncover more commonly mutated genes. (Price et al. [17] sampled ten colonies from four to seven tissue sites per patient in their VNTR-based study of acute melioidosis.) While *B. pseudomallei* strains have been shown to differ in their individual ability to cause disease [50], variation in the immune status and response of the infected human host clearly affects outcome [51]. These host differences may exert diverse selection pressures on the bacterium. It has been shown in patients deliberately colonized with asymptomatic bacteriuria *E. coli* that in addition to stochastic events, adaptive bacterial evolution was driven by individual host environments [52]. Additionally, since our isolate pairs were collected from different body sites, we cannot rule out the possibility that tissue-specific selection pressures exist.

Relapse has long been recognized as a potential problem in patients who survive acute melioidosis. While some clinical determinants of relapse are known, such as choice and duration of oral antimicrobial therapy [53], bacterial mechanisms contributing to persistent infections are poorly understood. Our results are consistent with a model involving adaptation during acute primary and relapse episodes with an intervening period of dormancy. Adaptive changes are often associated with increased antibiotic resistance, which may contribute to re-emergence. Indeed, documented relapse cases have been caused by isolates with acquired antibiotic resistance [54]–[56]. For patients who survive their primary episode, the single most serious complication is recurrent melioidosis after having an apparent cure [57]. As improved treatment results in a greater number of patients who survive primary infections, relapse will continue to be an important problem.

Sequencing platforms with low costs per raw base-call have made it practical to use deep sequence sampling to assess bacterial evolution in a clinical setting. The whole genome sequencing and analysis approaches used in our study provided the means for uncovering the extent of genomic diversity in B. pseudomallei clinical isolates that would have been difficult to describe by other methods. Further insight into recurrent melioidosis, as well as acute and chronic disease, will come from sequencing a larger set of sequential isolates from individual patients. Understanding the full extent of genetic changes in the pathogen as it infects and resides in the host should aid the development of better intervention strategies.

## Materials and Methods

### Bacterial Isolates and Genomic DNA Isolation

The ten *B. pseudomallei* isolates used for whole-genome comparison are listed in Table 1, and the 107 isolates investigated for BPSS1483 variants are listed in Table S6. Isolates were collected as described in Maharjan et al. [10]. Relapse was defined as the development of new symptoms and signs of infection in association with a positive *B. pseudomallei* culture after an initial response to therapy [52].

Genomic DNA for Illumina sequencing and SNP discovery in BPSS1483 homologs was isolated using the Gentra Puregene Yeast/Bact. Kit (Qiagen, Valencia, CA) according to manufacturer’s directions. The lysis step was conducted in the CDC select agent certified BSL3 containment facility at the University of Washington and the lysates were quarantined for 72 hours until sterility was confirmed.

### Whole-genome Sequencing of the *B. pseudomallei* 1026b Reference Strain

*B. pseudomallei* 1026b was sequenced to completion using standard capillary DNA shotgun sequencing protocols and data collection tools [58]. Sequences were assembled and viewed using Phred/Phrap/Consed software [59]–[61]. The quality and contiguity of the shotgun data were improved by three rounds of experiments designed by the Autofinish tool in Consed [62], followed by directed finishing experiments. The final gap in chromosome 1 was located in the third of the three rDNA operons. This gap was artificially closed by copying and inserting 5,344 bases from the upstream rDNA operon (2,556,685–2,562,028). The final gap in chromosome 2, which spanned the polyketide synthase locus PksL (BP1026B_II1104), was closed by independent assembly in the program Sequencher (Gene Codes, Ann Arbor, MI) of a 34 Kb contig composed of shotgun and directed sequencing reads from this region.

Both fosmid DNA fingerprints and Illumina short-read sequence data validated to base-pair accuracy the final 1026b genome assembly, which contained approximately 125,000 capillary sequencing reads. Fosmid fingerprints from EcoRI, MluI and PstI digestions were compared with the virtual sequence-derived fragments of the final assembly using sequence-validation features of Genomic Variation Analysis software [21]. Fingerprints for 1503 fosmids, providing nearly continuous 2X genome coverage of both chromosomes, corresponded to those of the final assembly. A total of 13 million paired-end 76 base reads were generated for the 1026b genome following manufacturer’s protocols (Illumina, San Diego, CA). Reads were mapped to the final sequence for the two 1026b chromosomes using MAQ [63] and discrepancies in the alignment were manually checked in Consed. The alignment, in which 99.78% of 1026b genome was covered by three or more reads, confirmed base calls from the capillary sequence data, except in regions with no Illumina read coverage.

### Detecting SNPs and Indels in 354a-354e, 1258a-1258b and 1026a

The genomes of 354a, 354e, 1258a, 1258b and 1026a were sequenced with the Illumina Genome Analyzer according to manufacturer’s instructions (Illumina, San Diego, CA). For each genome a random-fragment library was constructed using a custom paired-end protocol. Briefly, genomic DNA samples were sheared using a Biorupter UCD-200 (Diagenode Inc., Denville, NJ) and end-repaired. Repaired fragments were subjected to A-tailing using Taq DNA polymerase, and custom “Y” adaptors produced by hybridization of partially complimentary sequences were ligated to A-tailed fragments using T4 DNA ligase. Two adaptor oligonucleotides were employed: 1) PE_Adapt_hi (/5Phos/GATCGGAAGAGCGGTTCAGCAGGAATGCCGAG), and 2) PE_Adapt_lo (ACACTCTTTCCCTACACGACGCTCTTCCGATC*T), in which the asterisk indicates phosphorthiolation of the 3′-most base.

Reads for each isolate were mapped to the 1026b and K95243 reference genomes using the Burrows-Wheeler Aligner (BWA) program [64] in order to generate ungapped and gapped alignments for SNP and indel calling, respectively. SNPs were called using SAMtools [65]. SNPs in the raw pileup files for 354a and 354e and for 1258a and 1258b were compared to identify variants unique to the primary or relapse isolate in each pair. The list of unique SNPs for each strain was then filtered to require ≥5 reads and a SNP quality ≥10 and to eliminate positions with ambiguous consensus calls. Indels were called using the Genome Analysis Toolkit (GATK) whole genome, deep coverage pipeline with default parameters [66]. Prior to running GATK, reads were filtered for duplicates using Picard MarkDuplicates (http://samtools.sourceforge.net/) such that only the read pair having the highest sum of base qualities at any given start position and orientation was retained.

All resulting SNPs and indels were viewed in the Integrative Genomics Viewer v1.5 [67] to eliminate false positives. Selected variants were the major allele in one isolate of the clonal pair and were absent, or if present were the minor allele represented by <1% of reads, in the other isolate.

### Detecting SNPs, Indels and Reference Sequence Errors in 1106a-1106b and 1710a-1710b

Traces of Sanger reads for the 1106a, 1106b, 1710a and 1710b genomes were downloaded from the Trace Archive (www.ncbi.nlm.nih.gov/Traces/trace.cgi) and aligned to the complete, publicly-available 1106a or 1710b genomes using Phred/Phrap/Consed software. All high quality discrepancies and questionable consensus bases were manually checked and were included in the final count of SNPs and indels when a majority of reads (Q20 or greater) in the inter-isolate comparison, e.g. 1106b reads aligned to the 1106a genome, and no reads in the intra-isolate comparison, e.g. 1106a reads aligned to the 1106a genome, exhibited the SNP or indel. These criteria eliminated minor allele variants. Single base errors in the 1106a and 1710b reference sequences were identified when reads, and the underlying chromatograms, from neither the primary nor the relapse isolates agreed with the reference base (Table S5). We sequenced the 1710b isolate from our strain collection using the Illumina Genome Analyzer and aligned these reads to the 1710b reference sequence as described previously. The alignment confirmed all errors listed in Table S5.

### Structural Variation

Structural variants within clonal pairs of isolates were identified using large-insert genome analysis (LIGAN) technology based on a fosmid cloning strategy as described in Hayden et al. [21]. Briefly, deep fosmid libraries containing cloned inserts limited to approximately 32 to 48 Kb in length were constructed in the pCC1Fos vector (Epicentre, Madison, WI) for the isolates listed in Table 1. For all isolates except 1026b (described previously), fosmid end sequences and fingerprints using EcoRI, MluI and PstI restriction enzymes were generated for approximately 1200 randomly picked clones. Fosmid data for each isolate were analyzed independently using the Genomic Variation Analysis (GenVal) suite of software tools (http://depts.washington.edu/uwgcmed/software.html). GenVal determined positions for each clone with respect to a given reference sequence and identified locations of insertions, deletions and rearrangements based on inconsistencies with normal fosmid tiling. For example, fosmids spanning inversion breakpoints had paired-end sequences that did not point toward each other and deletions in the query genome were indicated when the apparent length of the anchored clone was greater than the physical clone insert length based on fingerprints (Figure 2). Fosmids for 1026a, 1106b and 1710a were tiled against their complementary reference genomes for direct primary-relapse strain comparison. Fosmids for the 354 and 1258 pairs were tiled against the 1026b and K96243 reference sequences and variants unique to either the primary or relapse isolate for each pair were identified.

### Validation and Annotation of SNPs, Indels and Structural Variation

All selected SNPs and indels were validated using Sanger sequencing of PCR products (not shown). Structural variants were confirmed and further characterized by shotgun sequencing of selected fosmid clones as previously described [58]. For NS SNPs, the effect of amino acid substitutions on protein function was assessed using SIFT [33], which predicts whether a substitution is tolerated based on sequence homology and the physical properties of the amino acids.

### SNP Discovery in BPSS1483 Homologs and Testing Antibiotic Susceptibility

The sequence of BPSS1483 was obtained for 107 *B. pseudomallei* strains (Table S6). The sequence was publicly available for eight strains. DNA for 87 strains was provided by Don Woods (University of Calgary, Alberta, Canada), and DNA for the remaining twelve strains was isolated as described above. The gene was PCR amplified and sequenced with the following primers: CGACGATTTTCTCGAAGG (forward) and CGATTCGATTCCACGGTA (reverse). PCR amplifications were performed using Phusion High-Fidelity DNA Polymerase and GC Buffer (New England Biolabs, Inc., Beverly, MA) and included 10% DMSO. After an initial denaturation step at 98°C for 30 sec, 30 cycles of denaturation at 98°C for 10 sec, annealing at 60°C for 30 sec and extension at 72°C for 30 sec were carried out followed by a final extension at 72°C for 5 min. Sequence reads were aligned to BPSS1483 and evaluated for SNPs in Sequencher (Gene Codes, Ann Arbor, MI). The effect of NS SNPs on protein function was assessed using SIFT [33].

Two *B. thailandensis* mutants were selected from a comprehensive, sequence-defined transposon mutant library (unpublished) for antibiotic susceptibility testing. The library was constructed in the reference *B. thailandensis* E264 strain using a conjugal plasmid bearing a T8 transposon, which carries a tetracycline resistance gene. Insertion sites were identified by PCR amplification and sequencing of one of the transposon-genome junctions. The test strain contained a transposon inserted into BTH_II0885, the homolog of the BPSS1483 TetR gene, following nucleotide 200 of 1023 total nucleotides of the coding region. This insertion is within the first fifth of the ORF and is thus likely to represent a null mutation. The control strain contained a transposon in the putative transposase gene BTH_I3273 and was used in place of the wild-type strain to compensate for any phenotypic effect of the antibiotic resistance gene carried by the transposon. The transposon insertion sites in the test and control strains were confirmed by re-sequencing single-colony purified cells.

Minimal inhibitory concentrations (MICs) were determined using Etest gradient strips (AB Biodisk, Piscataway, NJ) following manufacturer’s protocols. Assays of *B. pseudomallei* strains were conducted in the BSL3 containment facility at the University of Washington.

## Supporting Information

Table S1 Colors assigned to COG functional categories.
(XLS)

Table S2 Properties of strain 1026b compared to 13 publicly available *B. pseudomallei* genomes.
**(**XLS**)**

Table S3 Genes unique to or missing from the *B. pseudomallei* 1026b genome.
(XLS)

Table S4 Indels found in the 1106a-1106b, 1710a-1710b and 354a-354e isolate pairs.
(XLS)

Table S5 Errors in reference sequences for *B. pseudomallei* strains.
(XLS)

Table S6 List of *B. pseudomallei* strains included in TetR SNP analysis.
(XLS)

Table S7 Synonymous mutations in BPSS1483 homologs from 107 *B. pseudomallei* isolates.
(XLS)

Table S8 List of 17 genes in the 22 Kb tandem repeat of *B. pseudomallei* 1710a.
(XLS)

## References

[1] Dobrindt U, Zdziarski J, Salvador E, Hacker J (2010). Bacterial genome plasticity and its impact on adaptation during persistent infection.. Int J Med Microbiol.

[2] Smith EE, Buckley DG, Wu Z, Saenphimmachak C, Hoffman LR (2006). Genetic adaptation by Pseudomonas aeruginosa to the airways of cystic fibrosis patients.. Proc Natl Acad Sci U S A.

[3] Jelsbak L, Johansen HK, Frost AL, Thogersen R, Thomsen LE (2007). Molecular epidemiology and dynamics of Pseudomonas aeruginosa populations in lungs of cystic fibrosis patients.. Infect Immun.

[4] Hogardt M, Heesemann J (2010). Adaptation of Pseudomonas aeruginosa during persistence in the cystic fibrosis lung.. Int J Med Microbiol.

[5] Kraft C, Stack A, Josenhans C, Niehus E, Dietrich G (2006). Genomic changes during chronic Helicobacter pylori infection.. J Bacteriol.

[6] Oh JD, Kling-Backhed H, Giannakis M, Xu J, Fulton RS (2006). The complete genome sequence of a chronic atrophic gastritis Helicobacter pylori strain: evolution during disease progression.. Proc Natl Acad Sci U S A.

[7] Goerke C, Wolz C (2010). Adaptation of Staphylococcus aureus to the cystic fibrosis lung.. Int J Med Microbiol.

[8] Russell DG, Barry CE, Flynn JL (2010). Tuberculosis: what we don’t know can, and does, hurt us.. Science.

[9] Peacock SJ (2006). Melioidosis.. Curr Opin Infect Dis.

[10] Maharjan B, Chantratita N, Vesaratchavest M, Cheng A, Wuthiekanun V (2005). Recurrent melioidosis in patients in northeast Thailand is frequently due to reinfection rather than relapse.. J Clin Microbiol.

[11] Mays EE, Ricketts EA (1975). Melioidosis: recrudescence associated with bronchogenic carcinoma twenty-six years following initial geographic exposure.. Chest.

[12] Koponen MA, Zlock D, Palmer DL, Merlin TL (1991). Melioidosis.. Forgotten, but not gone! Arch Intern Med.

[13] Ngauy V, Lemeshev Y, Sadkowski L, Crawford G (2005). Cutaneous melioidosis in a man who was taken as a prisoner of war by the Japanese during World War II.. J Clin Microbiol.

[14] Galyov EE, Brett PJ, DeShazer D (2010). Molecular insights into Burkholderia pseudomallei and Burkholderia mallei pathogenesis.. Annu Rev Microbiol.

[15] Pearson T, U’Ren JM, Schupp JM, Allan GJ, Foster PG (2007). VNTR analysis of selected outbreaks of Burkholderia pseudomallei in Australia.. Infect Genet Evol.

[16] Sam IC, See KH, Puthucheary SD (2009). Variations in ceftazidime and amoxicillin-clavulanate susceptibilities within a clonal infection of Burkholderia pseudomallei.. J Clin Microbiol.

[17] Price EP, Hornstra HM, Limmathurotsakul D, Max TL, Sarovich DS (2010). Within-host evolution of Burkholderia pseudomallei in four cases of acute melioidosis.. PLoS Pathog.

[18] Nandi T, Ong C, Singh AP, Boddey J, Atkins T (2010). A genomic survey of positive selection in Burkholderia pseudomallei provides insights into the evolution of accidental virulence.. PLoS Pathog.

[19] Godoy D, Randle G, Simpson AJ, Aanensen DM, Pitt TL (2003). Multilocus sequence typing and evolutionary relationships among the causative agents of melioidosis and glanders, Burkholderia pseudomallei and Burkholderia mallei.. J Clin Microbiol.

[20] Holden MT, Titball RW, Peacock SJ, Cerdeno-Tarraga AM, Atkins T (2004). Genomic plasticity of the causative agent of melioidosis, Burkholderia pseudomallei.. Proc Natl Acad Sci U S A.

[21] Hayden HS, Gillett W, Saenphimmachak C, Lim R, Zhou Y (2008). Large-insert genome analysis technology detects structural variation in Pseudomonas aeruginosa clinical strains from cystic fibrosis patients.. Genomics.

[22] Kidd JM, Cooper GM, Donahue WF, Hayden HS, Sampas N (2008). Mapping and sequencing of structural variation from eight human genomes.. Nature.

[23] DeShazer D, Brett PJ, Carlyon R, Woods DE (1997). Mutagenesis of Burkholderia pseudomallei with Tn5-OT182: isolation of motility mutants and molecular characterization of the flagellin structural gene.. J Bacteriol.

[24] Thongdee M, Gallagher LA, Schell M, Dharakul T, Songsivilai S (2008). Targeted mutagenesis of Burkholderia thailandensis and Burkholderia pseudomallei through natural transformation of PCR fragments.. Appl Environ Microbiol.

[25] Propst KL, Mima T, Choi KH, Dow SW, Schweizer HP (2010). A Burkholderia pseudomallei deltapurM mutant is avirulent in immunocompetent and immunodeficient animals: candidate strain for exclusion from select-agent lists.. Infect Immun.

[26] Brittnacher MJ, Fong C, Hayden HS, Jacobs MA, Radey M (2011). PGAT: A multi-strain analysis resource for microbial genomes.. Bioinformatics.

[27] DeShazer D (2004). Genomic diversity of Burkholderia pseudomallei clinical isolates: subtractive hybridization reveals a Burkholderia mallei-specific prophage in B. pseudomallei 1026b J Bacteriol.

[28] Sim SH, Yu Y, Lin CH, Karuturi RK, Wuthiekanun V (2008). The core and accessory genomes of Burkholderia pseudomallei: implications for human melioidosis.. PLoS Pathog.

[29] Levinson G, Gutman GA (1987). Slipped-strand mispairing: a major mechanism for DNA sequence evolution.. Mol Biol Evol.

[30] U’Ren JM, Schupp JM, Pearson T, Hornstra H, Friedman CL (2007). Tandem repeat regions within the Burkholderia pseudomallei genome and their application for high resolution genotyping.. BMC Microbiol.

[31] Nierman WC, DeShazer D, Kim HS, Tettelin H, Nelson KE (2004). Structural flexibility in the Burkholderia mallei genome.. Proc Natl Acad Sci U S A.

[32] Song H, Hwang J, Myung J, Seo H, Yi H (2009). Simple sequence repeat (SSR)-based gene diversity in Burkholderia pseudomallei and Burkholderia mallei.. Mol Cells.

[33] Kumar P, Henikoff S, Ng PC (2009). Predicting the effects of coding non-synonymous variants on protein function using the SIFT algorithm.. Nat Protoc.

[34] Ramos JL, Martinez-Bueno M, Molina-Henares AJ, Teran W, Watanabe K, et al (2005). The TetR family of transcriptional repressors.. Microbiol Mol Biol Rev.

[35] Tuanyok A, Auerbach RK, Brettin TS, Bruce DC, Munk AC (2007). A horizontal gene transfer event defines two distinct groups within Burkholderia pseudomallei that have dissimilar geographic distributions.. J Bacteriol.

[36] Szklarczyk D, Franceschini A, Kuhn M, Simonovic M, Roth A (2011). The STRING database in 2011: functional interaction networks of proteins, globally integrated and scored.. Nucleic Acids Res.

[37] Haraga A, West TE, Brittnacher MJ, Skerrett SJ, Miller SI (2008). Burkholderia thailandensis as a model system for the study of the virulence-associated type III secretion system of Burkholderia pseudomallei.. Infect Immun.

[38] Schwarz S, West TE, Boyer F, Chiang WC, Carl MA (2010). Burkholderia type VI secretion systems have distinct roles in eukaryotic and bacterial cell interactions.. PLoS Pathog.

[39] Kumar A, Chua KL, Schweizer HP (2006). Method for regulated expression of single-copy efflux pump genes in a surrogate Pseudomonas aeruginosa strain: identification of the BpeEF-OprC chloramphenicol and trimethoprim efflux pump of Burkholderia pseudomallei 1026b.. Antimicrob Agents Chemother.

[40] Rholl DA, Papp-Wallace KM, Tomaras AP, Vasil ML, Bonomo RA (2011). Molecular investigations of PenA-mediated beta-lactam resistance in Burkholderia pseudomallei.. Front Microbiol.

[41] Chantratita N, Rholl DA, Sim B, Wuthiekanun V, Limmathurotsakul D (2011). Antimicrobial resistance to ceftazidime involving loss of penicillin-binding protein 3 in Burkholderia pseudomallei.. Proc Natl Acad Sci U S A.

[42] Sandegren L, Andersson DI (2009). Bacterial gene amplification: implications for the evolution of antibiotic resistance.. Nat Rev Microbiol.

[43] Liao X, Hancock RE (1997). Susceptibility to beta-lactam antibiotics of Pseudomonas aeruginosa overproducing penicillin-binding protein 3.. Antimicrob Agents Chemother.

[44] Wuthiekanun V, Peacock SJ (2006). Management of melioidosis.. Expert Rev Anti Infect Ther.

[45] Wuthiekanun V, Limmathurotsakul D, Chantratita N, Feil EJ, Day NP (2009). Burkholderia pseudomallei is genetically diverse in agricultural land in Northeast Thailand.. PLoS Negl Trop Dis.

[46] Pumpuang A, Chantratita N, Wikraipat C, Saiprom N, Day NP (2011). Survival of Burkholderia pseudomallei in distilled water for 16 years.. Trans R Soc Trop Med Hyg.

[47] Hamad MA, Austin CR, Stewart AL, Higgins M, Vazquez-Torres A (2011). Adaptation and Antibiotic Tolerance of Anaerobic Burkholderia pseudomallei.. Antimicrob Agents Chemother.

[48] Lillebaek T, Dirksen A, Vynnycky E, Baess I, Thomsen VO (2003). Stability of DNA patterns and evidence of Mycobacterium tuberculosis reactivation occurring decades after the initial infection.. J Infect Dis.

[49] Tribuddharat C, Moore RA, Baker P, Woods DE (2003). Burkholderia pseudomallei class a beta-lactamase mutations that confer selective resistance against ceftazidime or clavulanic acid inhibition.. Antimicrob Agents Chemother.

[50] Vesaratchavest M, Tumapa S, Day NP, Wuthiekanun V, Chierakul W (2006). Nonrandom distribution of Burkholderia pseudomallei clones in relation to geographical location and virulence.. J Clin Microbiol.

[51] Lazar Adler NR, Govan B, Cullinane M, Harper M, Adler B (2009). The molecular and cellular basis of pathogenesis in melioidosis: how does Burkholderia pseudomallei cause disease?. FEMS Microbiol Rev.

[52] Zdziarski J, Brzuszkiewicz E, Wullt B, Liesegang H, Biran D (2010). Host imprints on bacterial genomes–rapid, divergent evolution in individual patients.. PLoS Pathog.

[53] Limmathurotsakul D, Chaowagul W, Chierakul W, Stepniewska K, Maharjan B (2006). Risk factors for recurrent melioidosis in northeast Thailand.. Clin Infect Dis.

[54] Haase A, Melder A, Smith-Vaughan H, Kemp D, Currie B (1995). RAPD analysis of isolates of Burkholderia pseudomallei from patients with recurrent melioidosis.. Epidemiol Infect.

[55] Jenney AW, Lum G, Fisher DA, Currie BJ (2001). Antibiotic susceptibility of Burkholderia pseudomallei from tropical northern Australia and implications for therapy of melioidosis.. Int J Antimicrob Agents.

[56] Wuthiekanun V, Amornchai P, Saiprom N, Chantratita N, Chierakul W (2011). Survey of Antimicrobial Resistance in Clinical Burkholderia pseudomallei Isolates over Two Decades in Northeast Thailand.. Antimicrob Agents Chemother.

[57] Limmathurotsakul D, Peacock SJ (2011). Melioidosis: a clinical overview.. Br Med Bull.

[58] Wood DW, Setubal JC, Kaul R, Monks DE, Kitajima JP (2001). The genome of the natural genetic engineer Agrobacterium tumefaciens C58.. Science.

[59] Ewing B, Hillier L, Wendl MC, Green P (1998). Base-calling of automated sequencer traces using phred. I. Accuracy assessment.. Genome Res.

[60] Ewing B, Green P (1998). Base-calling of automated sequencer traces using phred. II. Error probabilities.. Genome Res.

[61] Gordon D (2003). Viewing and editing assembled sequences using Consed.. Curr Protoc Bioinformatics Chapter 11: Unit11.

[62] Gordon D, Desmarais C, Green P (2001). Automated finishing with autofinish.. Genome Res.

[63] Li H, Ruan J, Durbin R (2008). Mapping short DNA sequencing reads and calling variants using mapping quality scores.. Genome Res.

[64] Li H, Durbin R (2009). Fast and accurate short read alignment with Burrows-Wheeler transform.. Bioinformatics.

[65] Li H, Handsaker B, Wysoker A, Fennell T, Ruan J (2009). The Sequence Alignment/Map format and SAMtools.. Bioinformatics.

[66] McKenna A, Hanna M, Banks E, Sivachenko A, Cibulskis K (2010). The Genome Analysis Toolkit: a MapReduce framework for analyzing next-generation DNA sequencing data.. Genome Res.

[67] Robinson JT, Thorvaldsdottir H, Winckler W, Guttman M, Lander ES (2011). Integrative genomics viewer.. Nat Biotechnol.

